# Contribution of Resident Stem Cells to Liver and Biliary Tree Regeneration in Human Diseases

**DOI:** 10.3390/ijms19102917

**Published:** 2018-09-25

**Authors:** Diletta Overi, Guido Carpino, Vincenzo Cardinale, Antonio Franchitto, Samira Safarikia, Paolo Onori, Domenico Alvaro, Eugenio Gaudio

**Affiliations:** 1 Department of Anatomical, Histological, Forensic Medicine and Orthopedics Sciences, Sapienza University of Rome, Via Borelli 50, 00161 Rome, Italy; diletta.overi@uniroma1.it (D.O.); antonio.franchitto@uniroma1.it (A.F.); paolo.onori@uniroma1.it (P.O.); eugenio.gaudio@uniroma1.it (E.G.); 2 Department of Movement, Human and Health Sciences, Division of Health Sciences, University of Rome “Foro Italico”, Piazza Lauro de Bosis 6, 00135 Rome, Italy; 3 Department of Medico-Surgical Sciences and Biotechnologies, Sapienza University of Rome, Corso della Repubblica 79, 04100 Latina, Italy; v.cardinale80@gmail.com; 4 Department of Internal Medicine and Medical Specialties, Sapienza University of Rome, Viale del Policlinico 151, 00161 Rome, Italy; samira.safarikia@uniroma1.it (S.S.); domenico.alvaro@uniroma1.it (D.A.)

**Keywords:** hepatic stem/progenitor cells, biliary tree stem/progenitor cells, liver regeneration, liver fibrosis, cholangiopathies, peribiliary glands, ductular reaction

## Abstract

Two distinct stem/progenitor cell populations of biliary origin have been identified in the adult liver and biliary tree. Hepatic Stem/progenitor Cells (HpSCs) are bipotent progenitor cells located within the canals of Hering and can be differentiated into mature hepatocytes and cholangiocytes; Biliary Tree Stem/progenitor Cells (BTSCs) are multipotent stem cells located within the peribiliary glands of large intrahepatic and extrahepatic bile ducts and able to differentiate into hepatic and pancreatic lineages. HpSCs and BTSCs are endowed in a specialized niche constituted by supporting cells and extracellular matrix compounds. The actual contribution of these stem cell niches to liver and biliary tree homeostatic regeneration is marginal; this is due to the high replicative capabilities and plasticity of mature parenchymal cells (i.e., hepatocytes and cholangiocytes). However, the study of human liver and biliary diseases disclosed how these stem cell niches are involved in the regenerative response after extensive and/or chronic injuries, with the activation of specific signaling pathways. The present review summarizes the contribution of stem/progenitor cell niches in human liver diseases, underlining mechanisms of activation and clinical implications, including fibrogenesis and disease progression.

## 1. Introduction

**Figure 1:**
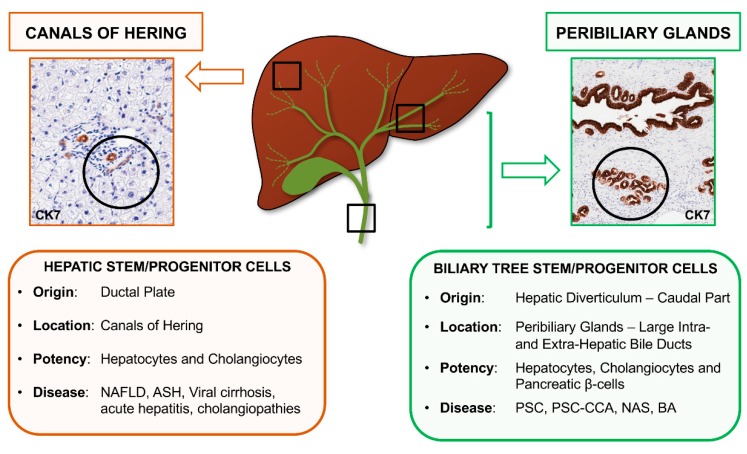
Stem/progenitor cell niches in the human biliary tree. Canals of Hering harbor Hepatic Stem/progenitor Cells (HpSCs), while peribiliary glands (PBGs) constitute the niche for Biliary Tree Stem/progenitor Cells (BTSCs). Embryological origin, location, potency, and diseases in which cells are involved are summarized in the boxes. CK7: cytokeratin 7; NAFLD: Non-alcoholic fatty liver disease; ASH: Alcoholic steatohepatitis; PSC: Primary sclerosing cholangitis, CCA: cholangiocarcinoma; NAS: non-anastomotic strictures; BA: biliary atresia. Original Magnification: 10× (left) and 5× (right).

In the adult liver and biliary tree, two distinct stem/progenitor cell populations have been described, namely Hepatic Stem/progenitor Cells (HpSCs) and Biliary Tree Stem/progenitor Cells (BTSCs). HpSCs are located in the smallest branches of the biliary tree (i.e., canals of Hering and bile ductules), while BTSCs are found in the peribiliary glands (PBGs) of large intrahepatic and extrahepatic bile ducts (Figure 1) [1,2]. Remarkably, mature parenchymal cells (i.e., hepatocytes and cholangiocytes) are characterized by high proliferative capabilities, which support their physiological turnover. Mature hepatocytes can undergo several rounds of replication but, with cellular aging and senescence, the majority of these cells becomes polyploid, have shortened telomeres, and suffer chromosomal alterations, which determine an impairment of cell replicative capability [3]. However, hepatocyte subpopulations with high replicative rates have been identified, and they take part in the physio-pathological renewal of liver parenchyma. Around the centrilobular vein, diploid Axin2+ hepatocytes are characterized by self-renewal properties and their progeny, during homeostasis, generate around 40% of hepatocytes [4]. Moreover, in a mouse model of liver damage, a population of so-called “hybrid hepatocytes” emerges and participates in liver renewal after damage, and is characterized by the expression of low levels of biliary-associated genes [5]. Furthermore, throughout the liver lobule, hepatocytes characterized by high expression of telomerase have been demonstrated to participate in the renewal of liver parenchyma; the maintenance of telomerase activity ensures the preservation of cellular replicative potential and genomic stability [6]. Cholangiocytes are also endowed with proliferative capabilities. The so-called “small cholangiocytes” have been particularly identified in experimental models, which constitute a subpopulation highly capable of proliferating in response to several physiological and pathological stimuli [7,8]. Moreover, the plasticity of both hepatocytes and cholangiocytes can account for tissue repair in the liver and biliary regeneration [9]. In mouse models, hepatocytes can transdifferentiate into mature cholangiocytes and form bile ducts that are effective in draining bile [10]. Conversely, in rodent models of regeneration, cholangiocytes can represent a source of new hepatocytes and gain a bi-phenotypic state in periportal regions and fibrotic septa [11].

In this light, the contribution of resident stem cell populations to the renewal of liver parenchyma remained an open question, and controversial evidence is present in the literature [3,9,12]. Indeed, studies in rodents have excluded the possibility that the resident stem/progenitor cell compartment contributes to the physiological turnover of mature hepatocytes [13,14,15].

More recently, extensive proliferation of HpSC pool has been demonstrated in experimental settings that determined large-scale injury and impairment of hepatocyte regenerative potentiality, with a relevant contribution of stem/progenitor cells in bile ductules to the restoration of liver integrity [11,16,17]. In keeping with this finding, following severe injuries induced by long-term exposure to toxic agents in mice, a large fraction of the liver parenchyma was replenished by bi-phenotypic cells derived from bile ductules in periportal regions and fibrotic septa [11]. On the other hand, BTSCs have been shown to contribute to the renewal of extrahepatic biliary tree in mice after experimentally induced damaging [18]. In summary, findings obtained in rodents suggested that a significant role for stem cell populations in liver physiological renewal could be excluded; however, severe damage and deterioration of mature cell function are compensated by the activation of stem/progenitor cell niche within the biliary tree.

Actually, experimental models in rodents often fail to faithfully reproduce human liver diseases [1]; in contrast to animal models, human liver diseases are usually characterized by long-term (i.e., chronic) exposure to damage and pathogenetic insults, accompanied by increased apoptosis and senescence [19,20,21] with severe impairment of regenerative capabilities of mature cells [22]. Accordingly, the activation of stem cell populations has been described in all human liver and biliary pathologies, and has a critical role in fibrogenesis, progression to cirrhosis, clinical manifestations, and prognosis [1,23].

The present review aimed to summarize the contribution of stem/progenitor cell niches in the course of human liver disease, to discuss the mechanisms and pathways of activation in disease pathogenesis and progression, and to disclose the most compelling clinical implications.

## 2. Recognizing Hepatic Stem/Progenitor Cells (HpSCs) and Their Progeny in Human Liver

**Figure 2:**
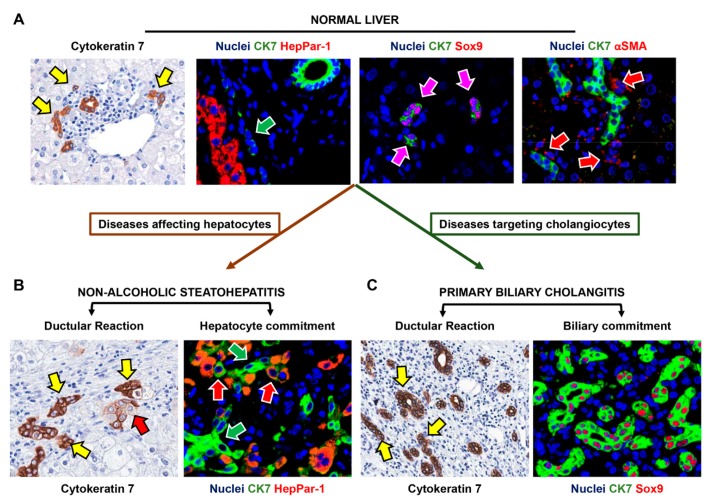
Hepatic Stem/progenitor Cell (HpSC) phenotype in normal liver and in pathological conditions. In normal liver (**A**), HpSC are recognizable (yellow arrows) thanks to the expression of cytokeratin 7 (CK7) and are located at the interface with hepatocyte plate as showed in double immunofluorescence (green arrows) with hepatocyte marker (HepPar-1). HpSCs express the stem cell marker Sox9 (purple arrows) and are surrounded by α-Smooth Muscle Actin (αSMA)+ myofibroblast-like cells (red arrows). In human disease affecting hepatocytes (**B**) as in non-alcoholic steatohepatitis, activation of HpSCs determines the appearance of ductular reaction (DR) with signs of hepatocyte commitment. In immunohistochemistry for CK7, DR (yellow arrows) is in close connection with hepatocyte plate and intermediate hepatocytes are present (red arrows); in double immunofluorescence, DR (green arrows) contains cells expressing HepPar-1 (red arrows). In diseases targeting cholangiocytes (**C**) as in primary biliary cholangitis, DR is prominent, located inside fibrotic septa (arrows), and composed of cells with an undifferentiated (Sox9)/biliary (CK7) phenotype. Original Magnification: 20× (immunohistochemistry) and 40× (immunofluorescence).

Hepatic Stem/progenitor Cells (HpSCs) are facultative bipotent progenitors, capable of differentiated into mature hepatocytes and cholangiocytes [24,25]. These cells are considered the remnants of the ductal plate, persisting in the adult liver as a population of Sox9+ cells within the Canals of Hering, as demonstrated by lineage tracing studies in mice (Figure 2) [2,26,27].

HpSCs are hardly recognizable in human liver given their small size, scant cytoplasm, and phenotype traits shared with mature cells. Regarding immunophenotype, HpSCs can express conventional stem cell markers (e.g., Sox9, Lgr5, CD44, CD133, Epithelial Cell Adhesion Molecule—EpCAM, and Neural Cell Adhesion Molecule—NCAM), cholangiocyte (e.g., CK7, CK19) and hepatocyte (e.g., CK18) cytokeratins, and, occasionally, hepatocyte traits (e.g., low levels of albumin). Thus, in human tissue, they can be individuated only taking in consideration their position within bile ductules (and Canals of Hering) in combination with their immunophenotype (Table 1) [28,29].

The progeny of HpSCs is represented by cells with an intermediate phenotype, which progressively acquire mature traits. Committed precursors for biliary cells progressively lose stem cell and neuroendocrine markers (e.g., CD133 and Lgr5) and do not express functional cholangiocyte markers (e.g., CFTR, AE2). On the other side, commitment toward hepatocyte fate implies the acquisition of an intermediate phenotype (so-called intermediate hepatocytes: IHs), which is characterized by an intermediate size between HpSCs and mature hepatocytes and by the expression of biliary cytokeratins and EpCAM [24,29]. Notably, HpSC behavior is dependent on the other cellular components of the niche, namely portal myofibroblasts, hepatic stellate cells and Kupffer cells, which provide the appropriate signals and sustain HpSC population both in quiescence and activated state (Figure 2) [30]. In general, HpSC activation is associated with the appearance of the so-called ductular reaction (DR) [31]. DR has been revealed in both acute and chronic human liver diseases and is constituted of proliferating strings of cells (i.e., reactive ductules) ranging from well-defined ducts to twisting structures without a distinct lumen. DR is composed of cells with a heterogeneous phenotype and highly variable profile, which is influenced by the regenerative needs due to the specific disease etiology [27,29]. In case of chronic biliary damage, DR is characterized by the proliferation of small cells expressing biliary traits, and stem cell/neuroendocrine markers (such as NCAM, Sox9 and CD133) [22,29] (Figure 2); differently, in liver diseases affecting hepatocytes, DR cells mostly show phenotypic traits of hepatocytes, with the appearance of numerous intermediate hepatocytes (Figure 2) [22,29].

## 3. HpSCs in Human Liver Diseases Targeting Hepatocytes

Ductular reaction has been described in a wide range of human pathologies that primarily target hepatocytes and is recognized as part of tissue response to insults, irrespective of disease etiology [32]. Prolonged and chronic injury to hepatocytes can progressively induce senescence, cell cycle arrest and impairment of the abovementioned proliferative capabilities, thus triggering the recruitment of the otherwise quiescent HpSCs [29,33,34]. Intermediate hepatocytes appear at the periphery of DR and in contiguity with proliferating HpSC [35] and can be recognized for their intermediate size between HpSCs and hepatocytes, and by their peculiar immunophenotype: They retain positivity for HpSC markers (e.g., CK7/19 and EpCAM) do not express mature cholangiocyte markers (e.g., AE2 or CFTR), and express hepatocyte markers (such as Hepatocyte Paraffin-1: HepPar-1, and albumin) [33,35,36].

The cellular source of DR could be represented not only by HpSC but also by mature cholangiocytes and mature hepatocytes, thanks to their plasticity [32]. Thus, in human diseases, it is not possible to exclude the derivation of IHs from mature cholangiocytes; however, their phenotype (lack of mature cholangiocyte traits and expression of HpSC traits) and their emergence also in cholangiopathies, where mature cholangiocytes undergo chronic inflammatory attack, would suggest their derivation from HpSC instead of mature cholangiocytes [33]. The observation within DR of HepPar-1+/CK19+ cells has been interpreted as signs of trans-differentiation (i.e., plasticity) of hepatocytes towards a ductular phenotype [37]; however, evidence in human pathology seems to suggest that the presence of HepPar-1+/CK19+ cells actually represents a sign of DR commitment toward hepatocyte fate. Indeed, in human liver diseases, HepPar-1+/CK19+ cells and EpCAM+ hepatocytes are not present at early stages, while DR and HpSCs can be observed before IHs, thus suggesting their derivation from this expanding cell compartment [35,38]. Moreover, the study of telomere lengths demonstrated that EpCAM+ hepatocytes display longer telomeres compared to mature hepatocytes, but shorter telomeres compared to DR cells, confirming that HpSC emergence precedes the appearance of intermediate hepatocytes [35]. Interestingly, studies of cirrhotic livers of different etiologies disclosed how areas of parenchymal extinction are repopulated by progenitors expanding from fibrous tracts via the sequence of hepatocyte bud formation, subsequently giving rise to new cirrhotic nodules [33,38]. The absence of hepatocytes in these areas prior to the formation of hepatocyte buds excludes a contribution of mature cells to this type of regeneration, which appears to be derived from HpSCs [33]. Nonetheless, the abnormal architecture of this type of cirrhotic nodules, characterized by portal vein branches at the center of the lobule, and hepatic vein and arteries in fibrotic tracts, compromises the capability of the liver to reconstitute normal functionality [38].

Ductular reaction has been extensively studied in human Non-Alcoholic Fatty Liver Disease (NAFLD), in which it has been correlated with the severity of damage and the progression of liver disease. NAFLD represents an increasing burden for western countries, often progressing from simple steatosis (i.e., fat accumulation in hepatocytes) to steatohepatitis, with extensive inflammation and fibrosis, representing one of the main causes for liver cirrhosis. In this context, a prominent DR characterizes both adult and pediatric patients affected by a more advanced disease. In such cases, hepatocyte cell cycle arrest and apoptosis induce the development of DR and the emergence of intermediate hepatocytes [19,39]. Interestingly, there is a strict correlation between DR extension and the entity of portal fibrosis and inflammation. In fact, HpSC proliferation is associated with the expansion of the other cellular components of the niche, which also take part in DR by furnishing several signals to proliferating and differentiating progenitors; this response is also fueled by the complex inflammatory and damage-associated milieu that characterizes severe and/or chronic human conditions. In this context, the processes aimed to achieve an adequate parenchymal regeneration are eventually overwhelmed, causing the progression of the disease by increasing collagen deposition and fibrosis and by the activation and recruitment of the macrophage populations [19,34,39,40]. 

Moreover, the HpSC niche activation has a proper role in influencing the clinical spectrum of NAFLD, independently of the severity of hepatocyte damage [41]. This aspect is especially evident when the relationship between DR and clinical features of NAFLD is taken into account. Pediatric NAFLD patients suffering from Obstructive Sleep Apnea Syndrome (OSAS) are characterized by higher activation of HpSC niche, with nocturnal hypoxemia being an independent predictor of HpSC activation [42]. Moreover, a peculiar pattern of HpSC niche activation can be observed in patients carrying the PNPLA3 I148M variant. Polymorphism in PNPLA3 gene have been proved to determine a more severe and progressive course of liver diseases of different etiologies. Particularly in NAFLD patients, the presence of PNPLA3 variant was associated with a more prominent DR and recruitment of cellular components of the niche (i.e., activated myofibroblasts and pro-inflammatory macrophages), independently of the disease grade and stage [41].

Interestingly, therapies that are able to improve liver histology in NAFLD patients have also an effect on the HpSC niche. The administration of docosahexaenoic acid (DHA, a polyunsaturated fatty acid) has been proved to modify liver macrophage activation state and cytokine milieu, correlated with the reduction of HpSC activation and improvement of NAFLD histological severity [43,44,45]; the administration of Vitamin D together with DHA in pediatric NAFLD patients led to the further reduction in myofibroblast activation and fibrogenesis in correlation with the histological depiction [45]. Moreover, in obese patients affected by NAFLD, when lifestyle or dietary measures do not lead to successful weight loss and clinical improvement, bariatric surgery could represent a therapeutic option to achieve long lasting beneficial effects. Interestingly, in NAFLD patients that underwent laparoscopic sleeve gastrectomy, the amelioration in disease stage and grade was associated with the reduction of hepatocyte senescence, DR extent and recruitment of cellular components of the niche [46].

Besides chronic conditions, severe acute injury can trigger HpSC proliferation in an attempt to rescue the sudden extensive hepatocyte loss. However, acute hepatitis, and especially acute liver failure (formerly known as “fulminant hepatitis”) are characterized by a prominent proliferation of HpSCs without relevant signs of differentiation towards hepatocyte fate, probably due to the altered signaling in the inflammatory milieu [29,47]. Interestingly, the extent of DR in fulminant hepatitis (e.g., acute liver failure) was associated with a negative prognosis, thus confirming the correlation between HpSC activation and disease severity in human pathology [48]. Alcoholic hepatitis complicates the course of alcoholic liver disease in heavy drinkers and is associated with high morbidity and mortality [49]; in these patients, HpSC compartment appears to be expanded, correlating with disease severity and predicting short-term mortality [49]. Clinically, a higher number of proliferating HpSCs has been observed in patients that respond to steroid therapy leading to a more favorable outcome [50], while “non-responders” are characterized by a limited capability of HpSCs to differentiate into hepatocytes, maintaining a biliary phenotype [51,52].

## 4. HpSCs in Human Liver Diseases Targeting Biliary Epithelium

Cholangiopathies are chronic diseases targeting the intrahepatic or extrahepatic biliary tree, resulting in the impairment of bile duct flow (cholestasis) and subsequent liver damage. Human cholangiopathies are often characterized by immune-mediated damage to bile ducts, with increased inflammatory infiltrate and fibrotic response. In this context, HpSC proliferation is triggered with the aim of supporting the renewal of mature cholangiocytes which are impaired in their proliferative capabilities by chronic damage [29].

Primary biliary cholangitis (PBC) and primary sclerosing cholangitis (PSC) are the two most common human cholangiopathies and differ in terms of primary target, histopathological features and clinical aspects. PBC is a chronic biliary disorder characterized by immune-mediated disruption of interlobular bile ducts, leading to ductopenia (i.e., the reduction of interlobular bile ducts per portal space). In contrast, PSC affects large intrahepatic and extrahepatic bile ducts, which display severe multiple strictures causing retrograde cholestasis and progressing to liver cirrhosis. In both diseases, a prominent DR appears, correlates with disease stage and fibrosis extent, and is associated with expanding stem/progenitor cells within developing fibrous septa [29]. However, according to the distinct pathogenetic insult and different site of primary injury, HpSC activation is characterized by a specific pattern in each disease: PSC is characterized by marked signs of hepatocyte fate commitment within DR and by the presence of numerous intermediate hepatocytes; differently, PBC-affected livers show a more prominent DR characterized by cells with a biliary phenotype [53].

The relationship between HpSC compartment and biliary fibrogenesis is also confirmed in pediatric cholangiopathies such as biliary atresia (BA) and Alagille syndrome. BA is characterized by the occurrence of severe stenosis of the extrahepatic bile ducts, which becomes clinically relevant in early post-natal days with severe cholestasis, requiring early surgical treatment and leading to liver failure, with need for liver transplantation in pediatric age for biliary cirrhosis. These patients are characterized by the emergence of DR, which correlates with an increasing fibrosis leading to progression of liver damage [54,55]. In contrast, Alagille syndrome is a congenital genetic disorder which, for a mutation in Jagged gene, causes severe impairment of bile duct formation and severe ductopenia. Interestingly, due to the altered Notch signaling, these patients do not display relevant DR and are characterized by low fibrosis extent, indicating that HpSC activation has a prominent role in driving fibrogenetic processes [54].

The strict association between HpSC niche activation and disease progression results in the correlation between DR and clinical aspects. In patients affected by chronic cholangiopathies, the extent of DR correlates with laboratory indexes of cholestasis (i.e., bilirubin in PBC and PSC, and γ-glutamyl transferase in PBC) and with prognostic risk scores (i.e., Mayo PSC score, UK-PBC risk scores, and Global PBC scores for transplantation-free survival) [53]. Moreover, in PBC patients, higher pre-treatment DR resulted in a lower chance of UDCA response, thus allowing early identification of patients needing second-line treatment options [56].

## 5. Supporting HpSC Response: The Niche and Signaling Pathways

HpSCs are located in a specialized anatomical and functional niche; HpSC expansion, proliferation and differentiation are strongly dependent on the supporting cells of the niche, the extracellular matrix composition, and the signaling pathways [1,53]. The cellular components of the HpSC niche are represented by portal myofibroblasts (MFs), hepatic stellate cells (HSCs) and resident macrophages (i.e., Kupffer cells), which are located within portal tracts and hepatic sinusoids [30].

A crucial element in hepatic regeneration after damage is the production of several humoral factors from the supporting niche cells [57]. Remarkably, the signaling pathways that take part in progenitor cell differentiation parallel the ones furnished during biliary and hepatic development. Diseases targeting biliary epithelium are characterized by the activation of Notch pathway within the niche; HSCs and MFs secrete Notch ligands (e.g., Jagged1), maintaining HpSCs in a biliary phenotype [29,53,58]. Conversely, the activation of WNT pathway drives HpSC proliferation and differentiation into mature hepatocytes, characterizing liver diseases in which hepatocytes are affected [44,53,57]; in such conditions, macrophages can produce WNT ligands and Tumor necrosis factor-like weak inducer of apoptosis (TWEAK), which is a potent inducer of HpSCs proliferation [59,60].

While HpSC activation and proliferation is guided and driven by other niche elements, HpSCs themselves produce signaling factors that concurrently regulate niche composition [30]; in this context, the expansion of HpSCs activates portal myofibroblast/HSC pool by secretion of Hedgehog (Hh) ligands [61], osteopontin, and TGF-β1; in pathological conditions, this results in the induction of collagen deposition [62,63] and leads to fibrogenesis and liver disease progression [31,64]. Interestingly, stimulation of Hh pathway in HpSCs themselves can trigger epithelial-to-mesenchymal transition (EMT), directly contributing to the myofibroblast pool [65,66]. 

Niche cellular components take part in the regulation of extracellular matrix (ECM) composition and continuous remodeling through the production of several metalloproteinases, allowing a fine-tuned maintenance of anatomical integrity of the niche [30,67]. HpSCs in particular are surrounded by a laminin-rich matrix, which furnishes signals needed to maintain an undifferentiated phenotype and proliferative state [68]; this interaction is promoted by β-galactoside-binding lectin galectin-3 [69] and mediated by NCAM expression by HpSCs [70]. Accordingly, PBC is characterized by an extensive laminin-rich matrix surrounding HpSCs, which reflects the prominent DR that takes place in PBC [53]. Conversely, cells leaving the laminin-rich niche are exposed to differentiative factors produced by niche elements, and thus undergo maturation towards hepatocytes [67,68]. In the course of liver injury, NCAM post-translational modifications (i.e., polySia) hamper cell-matrix interactions and facilitate HpSC migration from the niche, with subsequent differentiation [70]. Interestingly, diseases featuring higher expression of laminin within ECM (as in alcoholic hepatitis), are characterized by lower maturation of progenitors towards adult cells, which leads to inefficient repair of hepatic damage and worse outcome [51].

Generally, upon chronic injury, signals emanating from damaged tissue can induce stem cells to adopt behaviors that are not part of their homeostatic repertoire [71]; in liver human diseases, unappropriated signals within the niche maintain HpSCs in a proliferative state without the complete maturation towards a specific fate, thus leading to progressive ductular reaction and driving myofibroblast pool activation and progressive fibrosis [31]. Therefore, insights into niche orchestrators and the delineation of multifactorial determinants in HpSC behaviors would represent promising approaches in driving regenerative pathways in human liver diseases.

## 6. Biliary Tree Stem/Progenitor Cells (BTSCs)

**Figure 3:**
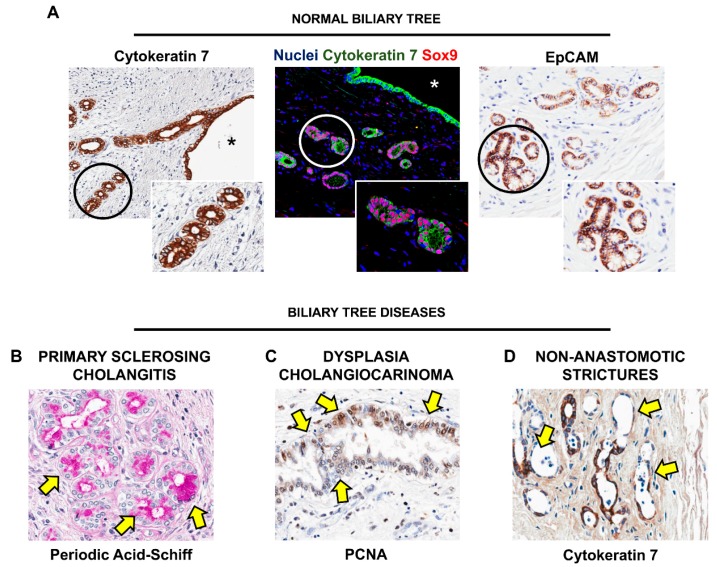
Biliary Tree Stem/progenitor Cells (BTSCs) and peribiliary glands (PBGs) in human biliary tree. PBGs are located in normal large intrahepatic and extrahepatic bile ducts (**A**) and are in direct communication with the bile duct lumen (asterisks). In PBGs, BTSCs can be identified by the positivity to stem cell markers, such as Sox9 and EpCAM. Circled areas are magnified in the boxes. In diseased ducts, such as Primary Sclerosing Cholangitis (**B**), dysplastic/neoplastic transformation (**C**), and non-anastomotic strictures (**D**), PBGs showed different degrees of mucinous metaplasia (arrows in **B**), proliferation (Proliferating Cell Nuclear Antigen: PCNA, arrows in **C**) and necrosis (arrows in **D**). Original Magnification: 10× (**A**) and 20× (**B**–**D**).

BTSCs are multipotent stem/progenitor cells, which are capable to give rise to mature cholangiocytes, hepatocytes, and pancreatic islets [72,73]. Their niche is represented by peribiliary glands (PBGs), which are tubulo-alveolar glands located within the wall of extrahepatic and large intrahepatic bile ducts (Figure 3) [74,75]. 

BTSCs can be found at the bottom of PBGs, and can be identified by the expression of specific stem cell markers (e.g., Sox17, Pdx1, Sox9, EpCAM, Sall4 and Lgr5) (Figure 3), while they usually lack markers of mature cells (e.g., secretin receptor—SR, albumin, insulin) [75]; a subpopulation of BTSCs expresses also markers which characterize pluripotent stem cells (i.e., Oct4, Sox2 and Nanog). In addition to the expression of stem cell markers observed in situ, the stemness of BTSCs has been also demonstrated in vitro. The self-renewal properties of BTSCs have been proven by culturing cells both on plastic and in Kubota’s Medium, a medium that allows the proliferation of endodermal stem cells but not of mature epithelial and mesenchymal cells: In such conditions, BTSCs are able to form colonies and can proliferate for months, maintaining an undifferentiated/stem-like phenotype (e.g., Sox9+/Sox17+/Pdx1+ and albumin-/SR-/insulin-) [72,73]. Moreover, BTSCs can differentiate in vitro towards a mature fate when cultured in media specifically tailored to provide the needed signals [72,73].

Evidence for a PBG network harboring proliferating progenitors has been also obtained in mice, where bile duct ligation has been shown to induce proliferation of Sox17+ and/or Pdx1+ cells, suggesting a role for these cells in epithelial renewal after damage [18]. Differentiative capabilities of BTSCs have been investigated in situ in murine experimental model, and in human pathologies. Both in experimentally-induced (streptozotocin administration in mice) and human diabetes, BTSCs within PBGs proliferate and show signs of maturation toward pancreatic lineage (i.e., insulin expression) [72,76]. Engraftment of BTSCs in immunocompromised (SCID) mice liver resulted in the appearance of hepatocytes expressing human antigens (e.g., human mitochondria and albumin) within mice hepatic parenchyma [77,78]; moreover, engrafted cells underwent proliferation after induction of liver injury by CCl_4_ administration, accompanied by an improvement in serological liver tests [79]. Intriguingly, results from a clinical trial using fetal BTSCs in cirrhotic liver patients show an improvement in liver biochemistry and clinical scores after BTSC infusion in the liver, possibly proving BTSC engraftment in human liver [80].

## 7. BTSC and Peribiliary Glands (PBG) Involvement in Human Pathologies

The involvement of PBGs in the pathogenesis and development of liver and biliary diseases is being increasingly acknowledged. PBGs are frequently affected in the course of pathological conditions of the hepatobiliary system, displaying pathological features comprising necro-inflammation, dilatations, along with hyperplasia and neoplastic transformation (Figure 3) [74].

A prominent involvement of PGBs and BTSCs has been observed in PSC. In this condition, large bile ducts are dramatically injured, with extensive inflammation and progressive fibrosis leading to clinically relevant strictures, chronic cholestasis and secondary liver parenchyma damage. In course of PSC, PBGs undergo massive proliferation and remodeling, characterized by the presence of hyperplastic/dysplastic glands and by an increased expression of stem/progenitor cell markers, suggesting the expansion of BTSC pool within glands [81]. Moreover, as the disease progresses, the BTSC niche appears to be diffusely and massively involved in PSC lesions, as chronic inflammatory and fibrogenetic response are increasingly sustained by the BTSC niche. Progressive injury triggers the reactive expansion of PBGs within bile duct walls, accompanied by the acquiring of a secretory phenotype by PBG cells, with production of pro-inflammatory and angiogenetic factors and recruitment of fibrogenetic cells. These modifications appear as a gradual spectrum of modifications in bile duct wall, progressing from inflammation, to duct wall thickening and strictures, which are accompanied by the appearance of dysplastic and, as the pathogenetic insult persists, neoplastic lesions within PBGs, leading to carcinogenesis and development of cholangiocarcinoma [82]. The appearance of lesions at a different stage in the same patient, with pre-neoplastic lesions affecting simultaneously several ducts, could represent a possible target for early diagnosis of cholangiocarcinoma in PSC patients [82].

Specific signaling pathways are involved in pathologic transformation of PBGs and the BTSC niche. In PSC ducts, the observed expansion of inflammatory/mesenchymal cell pool within PBGs is associated with an increased production of Hedgehog pathway ligands (i.e., Patched and Gli-1), driving the expression of EMT traits (i.e., Hh ligands, α-Smooth Muscle Actin—αSMA, Snai1 and Twist) in BTSCs, possibly leading to increased fibrogenesis [81,82]. Similarly, a role for Hedgehog pathway has been suggested in the development of stenotic lesions in biliary atresia: Interestingly, an increase in Hh ligands in PBG cells has been observed in extrahepatic bile ducts of patients affected by biliary atresia, which also correlated with poor jaundice-free survival [83].

PBG hyperplasia and dilatation have been observed in liver samples obtained from cirrhotic patients undergoing orthotopic liver transplantation (OLT); interestingly, these pathological lesions were accompanied by increased expression of EpCAM by PBG cells in dilated glands, together with the appearance of a CD133+ population [84]. Moreover, PBGs within bile ducts in samples obtained from patients affected by hepatolithiasis were characterized by increased expression of Sox9 and EpCAM, along with stem cell markers CD44s and CXCR4, compared to normal ducts [74].

In general, stem/progenitor cells are characterized by higher resistance to pathogenetic insults compared to mature cells [85]. Accordingly, bile ducts from explanted livers for OLT are characterized by extensive surface epithelium disruption, with relative preservation of PBGs and BTSCs, suggesting they are less sensitive to ischemia/reperfusion injury [86]. Non-anastomotic bile duct strictures (NAS) are a complication of OLT, characterized by severe stenosis of the bile ducts, leading to re-transplantation for chronic cholestasis; particularly, organ recipients from cardiac death donors are more prone to develop NAS, due to the longer ischemia time. In this context, it has been demonstrated that, in patients that later developed NAS, donor bile duct was characterized by extensive damage of PBGs, which were diffusely necrotic and associated with overall disruption and inflammation of bile duct wall, and impairment of peribiliary vascular plexus. These findings suggest how the integrity of PBGs and the BTSC pool could have a prominent role in the reconstitution of biliary epithelium and bile duct integrity after transplantation procedures [86,87].

## 8. Conclusions

The study of human pathologies demonstrated the relevant involvement of stem/progenitor cell niches in the development and progression of hepatic and biliary tree diseases; the involvement of these niches can be considered a marker of liver or biliary injury and an indicator of clinical and pathological severity. Furthermore, the balance between activation and differentiation of stem/progenitor cells together with the contribution of the other niche cells seems to be crucial in allowing the effective repair of damaged parenchyma. The modulation of signaling pathways within the niche could represent a target in order to guide stem/progenitor cell activation towards tissue repair, reducing pro-fibrogenetic signals and, thus, improving patient clinical outcomes.

## Figures and Tables

**Table 1:** Immunophenotype of Hepatic Stem/progenitor Cells (HpSCs) and their progeny in human liver.

Marker	Hepatocytes	Intermediate Hepatocytes	HpSCs	Immature Cholangiocytes	Cholangiocytes
CK7	−	+	+	+	+
CK19	−	+/−	+	+	+
EpCAM	−	+	+	+	−
Sox9	−	−	+	+	−
CD133	−	−	+	−	−
Lgr5	−	−	+	−	−
NCAM	−	−	+	+	−
Albumin	+	+	+/−	−	−
HepPar-1	+	+	−	−	−
HNF4α	+	+	−	−	−
CFTR	−	−	−	−	+
AE2	−	−	−	−	+
SCTR	−	−	−	−	+

CK: cytokeratin; EpCAM: Epithelial Cell Adhesion Molecule; NCAM: Neural Cell Adhesion Molecule; HepPar-1: Hepatocyte Paraffin 1; HNF: Hepatic Nuclear Factor; CFTR: Cystic Fibrosis Transmembrane Receptor; AE2: Anion Exchanger 2; SCTR: Secretin Receptor.
